# Comparative Morphometric and Structural Analysis of the Ovine Brain: Integrating Traditional Anatomical Methods with Artificial Intelligence-Driven 3D Modeling and Identification

**DOI:** 10.3390/vetsci13050447

**Published:** 2026-05-01

**Authors:** Moustafa Salouci

**Affiliations:** Department of Anatomy, College of Veterinary Medicine, King Faisal University, Al-Ahsa 36362, Saudi Arabia; msalouci@kfu.edu.sa

**Keywords:** veterinary anatomy, AI in anatomy, sheep brain, morphometrics, 3D reconstruction, neuroanatomy

## Abstract

The study of veterinary anatomy is gradually progressing with the combination of digital imaging and artificial intelligence (AI). This paper aimed to evaluate the potential use of AI tools for morphometric analysis and the anatomical identification of the ovine brain. Five adult specimens were used and approached through traditional dissection and fixation methods followed by digital photography and manual measurement with high-precision Vernier calipers. These results were compared against AI-based approaches, including DeeVid AI for 3D reconstruction, Imageonline for digital measurement, and ChatGPT/Artlist for anatomical nomenclature. The findings indicate that AI tools like DeeVid AI significantly enhance structural visualization, and Imageonline provides high-precision measurements comparable to manual tools (*p* > 0.05). However, AI-driven anatomical naming remains prone to significant errors, with ChatGPT and Artlist exhibiting error rates of 87.5% and 70.8%, respectively, in specific neuroanatomical labeling. This study concludes that while AI eases the reshaping and measurement of anatomical structures, human expertise remains indispensable for accurate anatomical identification.

## Simple Summary

This study explored how artificial intelligence (AI) can help in studying the anatomy of the sheep brain. Five adult sheep brains were examined using traditional methods like dissection, photography, and manual measurements, and then compared these results with AI tools. This study found that AI tools were very useful for creating clear 3D images and taking accurate measurements, with results similar to manual methods. However, AI performed poorly when identifying and naming brain structures, making many mistakes. Overall, the study shows that AI is helpful for visualization and measurement, but expert human knowledge is still essential for correctly identifying anatomical structures.

## 1. Introduction

The field of veterinary anatomy is very important to study clinical diagnosis, surgical aspects, and biological research. Accurate anatomical knowledge forms the basis of every aspect of veterinary medicine, from understanding normal structure to interpreting pathological changes in clinical aspects. Traditionally, the study of animal structures has been based on gross dissection and physical preservation techniques. However, the advancement of novel imaging technologies, including computed tomography (CT), magnetic resonance imaging (MRI), and X-ray radiography has deeply revolutionized our ability to visualize internal structures with high remarkable precision [1,2,3,4]. CT offers high-resolution cross-sectional imaging of bone and dense tissues [5,6,7], MRI provides excellent soft-tissue contrast for neuronal and parenchymal tissues [8,9], and X-ray continues to be a vital and readily available technique for skeletal evaluation in veterinary medicine [10]. In parallel, plastination has been developed as an advanced preservation technique to produce permanent, dry, and odorless specimens for both teaching and research [11,12]. Plastinated specimens have demonstrated clear pedagogical value in anatomy education [13]. Recent advances in CT and MRI imaging have been more closely integrated into plastination processes providing improved, multi-modal learning materials that provide the tactile authenticity of plastinated specimens with the numeric accuracy of digital images [14]. More recently, artificial intelligence (AI) has started to exert a transformative influence across all facets of anatomical science, offering new avenues for education, research, and diagnostic imaging [15,16,17].

The importance of AI (artificial intelligence) in education and research cannot be overstated. In veterinary medicine in particular, AI is swiftly evolving from an experimental novelty into a standard technological approach, facilitating activities ranging from automated image segmentation and morphometric analysis to the production of interactive three-dimensional models for use in the classroom [18,19]. Sahu et al. [18] confirmed that AI-assisted protocols can efficiently generate interactive 3D anatomical models based on photographic as well as radiographic data, significantly minimizing the time and knowledge needed for building digital models in veterinary anatomy. In terms of research, AI provides the means to interrogate large datasets consistently and quickly, at a scale that far surpasses what could be achieved through manual means, potentially allowing for population-level morphometric analyses that were once thought of as impractical. However, Bertin et al. [20] indicated that although AI holds great promise for accelerating veterinary research, it also introduces potential risks such as increasing biased training data, opaque decision-making processes, and even creating over-reliance that might erode fundamental analytical skills among researchers. These dual realities underscore the need for experimental studies that rigorously test AI performance in specific veterinary anatomical contexts.

The ovine brain shares structural complexity with the human brain, making the sheep a well-established large-animal model for investigating neurological conditions and human disease [21,22]. Sheep are increasingly being used in translational research on neurological disorders [22,23], and a One Health approach to their use in neuroscience research has been suggested to combine veterinary and human medicine [24]. The development of dedicated brain atlases and the mapping of sheep brain architecture to human homologs further highlight the importance of rigorous ovine neuroanatomy [23]. Thus, the accuracy of the anatomical investigation in this species is critically important for both veterinary medicine and biomedical translation.

Although the computing capabilities have advanced, calculating the anatomy surfaces and volumes is still often a time-consuming task. Traditional instruments such as the digital Vernier caliper produces high accuracy results, but it is limited to physical samples. There is a growing need for digital tools that can facilitate measurements on reshaped or reconstructed images. The addition of three-dimensional printed models has been proposed in this context, and multi-colored 3D printed models significantly enhance students’ success and motivation in courses of veterinary anatomy [25]. Virtual and comparative animal anatomy teaching across species—feline, equine, ovine and more—also presents a new frontier in “tech-friendly” anatomy education [26]. AI may provide the answer to make comparison easier between real anatomical images and AI reshaped 3D models, with positive implications in diagnostics, morphometric evaluation, and anatomical teaching [15,16,27]. Gayatri et al. [19] makes a powerful case for why AI should be positioned as a valuable complementary tool in veterinary anatomy education, one that augments, rather than replaces, traditional methods by offering on-demand visualization, interactive labeling, and personalized learning pathways. Choudhary et al. [16] similarly provide a broad exploration of both the potential and limitations of AI in veterinary anatomy, emphasizing that although AI enhances efficiency and accessibility, its integration should be accompanied by critical evaluation and expert supervision.

Nevertheless, a few key questions remain regarding the accuracy of AI in species-specific anatomical nomenclature. Severe limitations for AI-based recognition have been reported in recent human anatomical studies: ChatGPT achieved only approximately 54% accuracy when naming specific anatomical structures from images [28] and provided just 57.3% correct answers to dichotomous clinical anatomy questions [29], while general AI image-interpretation tasks reached an average of 68% accuracy to identify structures [30]. Performance benchmarking has also revealed variable accuracy trends across different AI model generations in anatomy education contexts [31]. Parallel assessments of AI responses to anatomy examination questions have reported similar modest scores [32]. Concerns about AI-generated labeling errors and inaccurate anatomical image generation have been similarly raised across the literature [33].

The near-complete lack of species-specific veterinary neuroanatomical data in publicly accessible AI training datasets means that current AI models are in fact used as out-of-domain models when faced with ovine brain structures, a gap this study directly exposes and quantifies. Ethical dimensions of AI deployment in veterinary medicine, including concerns about accountability, data provenance, and the risk of perpetuating errors in clinical and educational settings, have also recently come to the fore [20], making rigorous evaluation studies such as this one all the more timely. The potential role of large language models and AI image tools in veterinary anatomy education is therefore best understood not as a replacement for expert knowledge, but that of a field where capabilities and limits should be carefully empirically assessed [16,19,20].

The present study holds rarity and significance within veterinary medicine: as far as the authors are aware, this is one of the first investigations to compare AI-derived morphometric measurements and anatomical nomenclature against traditional expert methods specifically on ovine neuroanatomical specimens.

## 2. Materials and Methods

**Figure 1:**
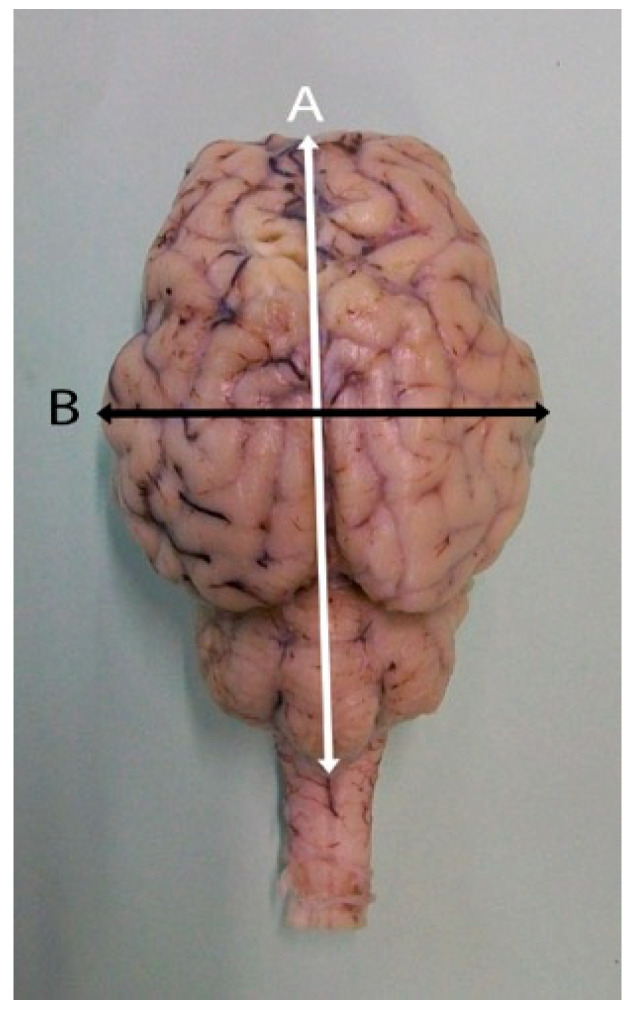
Dorsal view of an adult sheep brain illustrating the methodology for manual morphometric assessment. Brain length (**A**) was measured along the longitudinal axis from the apex of the cerebral hemispheres to the caudal end of the vermis. Brain width (**B**) was measured at the widest transverse point across the cerebral hemispheres.

Five heads of adult sheep were used in the study; they were obtained from a local slaughterhouse in the Kingdom of Saudi Arabia. All brains were harvested in the dissection hall following a standardized protocol. The heads were placed on a dissection table, and the skin and underlying soft tissues were removed to visualize the cranium. The mandible was disarticulated to facilitate access. For the extraction, the frontal bone was trepanned using an oscillating saw. After removing a portion of the meninges, the whole head was immersed in a 10% formalin solution for a period of ten days to ensure proper fixation. Every three days, the cranial opening was enlarged, and the head was re-immersed until the brain could be completely removed. All specimens were examined to ensure the absence of malformations or pathological lesions. The fixed brains were carefully examined, and the anatomical structures were identified and named in accordance with the Nomina Anatomica Veterinaria [34]. Digital images were obtained with a 6-megapixel Sony DSC-W50 camera (Group Corporation, Tokyo, Japan). Manual measurements of the brain length (from the apex of the cerebral hemisphere to the caudal end of the vermis) and width were recorded using a digital Vernier caliper (Necessities-Mall) with an accuracy of 0.01 mm (Figure 1).

Four AI tools were used to evaluate anatomical analysis in this study. DeeVid AI (v1.0) was used together with ChatGPT (v5.3) to convert 2D digital images into 3D aspects, enhancing structural depth and visualization. Imageonline.io (Free Online Image Measurement Tool) was used to perform linear measurements on the captured photographs for comparison with physical caliper data. Toolkit.artlist.io and ChatGPT were also used to identify and name specific anatomical structures based on standardized prompts. None of these tools were specifically calibrated or pre-trained by the authors; all were used as freely available, general-purpose web tools. The methodology for AI-assisted anatomical identification followed a standardized, multi-step protocol. First, high-resolution digital images of each brain specimen were acquired using a Sony DSC-W50 camera. Second, the images were uploaded directly to each AI platform via their respective web interfaces. Third, a consistent and standardized prompt was applied across all identification tasks, for example: “Identify the neuroanatomical structures in this dorsal view image of an adult sheep brain according to Nomina Anatomica Veterinaria”. Fourth, the AI-generated outputs, including labeled structures, were recorded verbatim without modification. Finally, all outputs were independently evaluated by two experts in veterinary anatomy, who assessed the accuracy of each labeled structure in accordance with the Nomina Anatomica Veterinaria [34].

All quantitative data were analyzed with SPSS (version 25.0). Descriptive statistics, including means and standard deviations (Mean ± SD) were computed for both real (caliper) and AI (Imageonline) measurements of the cerebrum. A paired *t*-test was performed to determine whether there was a systematic difference between the two methods of measurement. Statistical significance was defined as *p* < 0.05.

## 3. Results

### 3.1. 3D Structural Transformation

**Figure 2:**
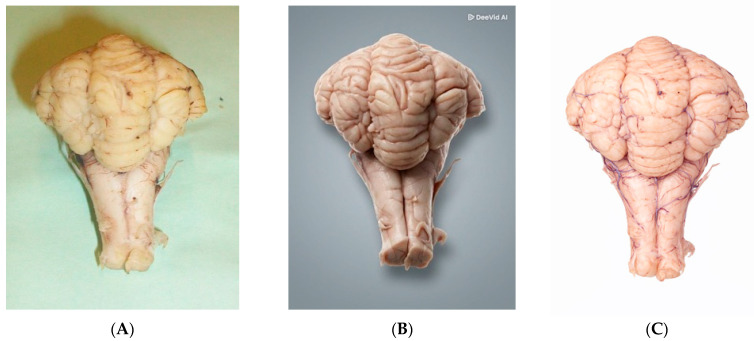
Comparative visualization of the real cerebellum (**A**) and the AI-transformed 3D models: (**B**) DeeVid AI stylized visualization (enhanced surface texture and depth for educational illustration); (**C**) ChatGPT conceptual schematic reconstruction (diagrammatic approximation of 3D structure).

Converting 2D images into 3D models using DeeVid AI provided a clearer visualization of the cerebellum’s folia and the cerebrum’s gyri (Figure 2). The AI-reshaped images allowed for a better spatial understanding of the sulci compared to static 2D photographs. Notably, DeeVid AI produced a stylized 3D visualization enhancing the texture, depth, and surface detail of the brain in a visually enriched manner suited for educational illustration, whereas ChatGPT generated a more conceptual schematic reconstruction, offering a diagrammatic approximation of the 3D structure rather than a photorealistic model. These two approaches thus serve distinct purposes: DeeVid AI is better suited for visual teaching aids, while ChatGPT provides a conceptual overview that may be useful for introductory-level orientation.

### 3.2. Morphometric and Anatomical Nomenclature Comparison

The comparative analysis between manual measurements (Real) and AI-based measurements (Imageonline) showed high levels of consistency. Table 1 summarizes the dorsal brain measurements for five samples.

**Figure 3:**
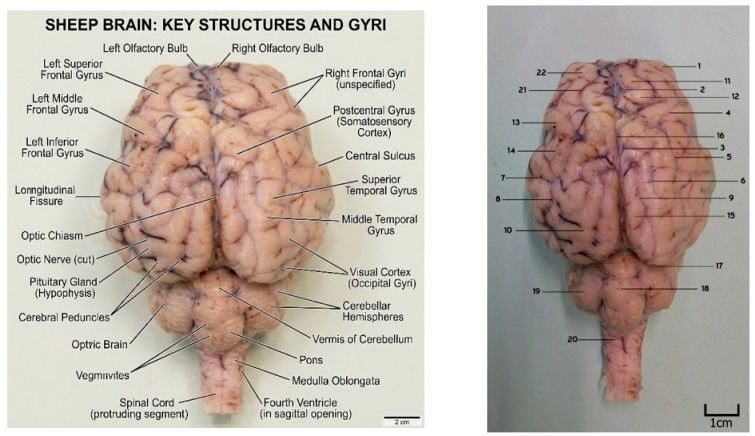
Dorsal view of the sheep brain: AI-labeled brain structures using Imageonline (**left**) compared with expert anatomical identification (**right**). Notably, numerous errors were observed in the labeling of structures when using the AI tool compared to the expert-based identification. 1. Prorean gyrus, 2. Cruciate sulcus, 3. Ansate sulcus, 4. Rostral suprasylvian sulcus, 5. Middle suprasylvian sulcus, 6. Marginal sulcus (sagittal sulcus), 7. Oblique sulcus, 8. Caudal suprasylvian sulcus, 9. Endomarginal sulcus, 10. Ectomarginal gyrus, 11. Precentral gyrus, 12. Postcentral gyrus, 13. Rostral ectosylvian gyrus, 14. Middle ectosylvian gyrus, 15. Marginal gyrus, 16. Longitudinal fissure of the cerebrum, 17. Transverse fissure of the cerebrum, 18. Vermis (of cerebellum), 19. Cerebellar hemisphere, 20. Medulla oblongata, 21. Coronal sulcus, 22. Rostral ectomarginal gyrus.

The identification of key structures such as the olfactory bulb, longitudinal fissure, and optic chiasm was attempted using AI labeling tools. However, the AI tools exhibited significant inaccuracies in identifying specific veterinary sulci and gyri. Common errors included mislabeling the cruciate sulcus as the central sulcus (a human anatomy term) and failing to correctly identify the prorean gyrus or specific cerebellar lobules such as the declive and culmen (Figure 3).

## 4. Discussion

The integration of AI into veterinary anatomy presents three distinct and interrelated areas of impact: visualization, quantification, and identification. The present study, which represents one of the few rigorous empirical assessments of AI performance on ovine neuroanatomical material, provides a detailed picture that clearly defines the strengths and limitations of AI. A balanced consideration of both the positives and negatives of AI in this domain is essential for guiding its responsible and effective adoption in veterinary medicine and anatomical research. Transforming 2D anatomical sections into 3D images via DeeVid AI offers substantial advantages for educational and research purposes. It allows for the analysis of structural depth that is often lost in standard photography. As seen in the results, the cerebellum’s complex folding becomes more apparent, aiding in the study of neuroanatomical spatial relationships. This aligns with the broader consensus that AI-driven visualization and 3D reconstruction significantly improve learning outcomes and spatial comprehension in veterinary and medical education [15,16]. The positive role of AI in generating interactive 3D anatomical models has been specifically validated in a veterinary anatomy context by Sahu et al. [18], who demonstrated that AI-assisted workflows could produce highly accessible, interactive models suitable for student use with minimal specialist input. This is particularly significant for institutions with limited access to cadaveric material, where AI-generated models may serve as a primary educational resource. AI-assisted imaging techniques have proven their ability to support more efficient and non-destructive analysis of anatomical specimens across a range of veterinary diagnostic imaging contexts [17,27]. These developments complement non-AI advances in anatomy teaching, such as plastination, a method whose comparative effectiveness versus formalin-fixed cadavers and digital 3D models continues to be actively evaluated [11]. The availability of plastinate libraries as dedicated educational repositories has further enriched the resources available to anatomy students and instructors. The integration of CT and MRI imaging with plastination processes, as documented by Alcobaça et al. [14], exemplifies the synergistic potential of combining traditional anatomical preservation with modern imaging modalities. Furthermore, the establishment of low-budget plastination laboratories has also made these techniques more accessible to institutions with limited resources [12], and the combination of such approaches with AI tools represents a promising direction for democratizing high-quality anatomical education globally.

The statistical comparison (Table 1) demonstrates that digital measurement tools such as Imageonline are highly reliable. The mean brain length recorded using the manual caliper was 8.48 ± 1.02 cm compared to 8.48 ± 1.04 cm via AI-assisted digital measurement, a negligible difference confirmed by a paired-samples *t*-test (*p* = 0.8914). Similarly, the mean cerebrum width measured 5.58 ± 0.64 cm manually versus 5.51 ± 0.76 cm digitally (*p* = 0.5982). Neither comparison approached statistical significance, providing preliminary evidence that AI-assisted image measurement may offer accuracy comparable to physical calipers for this type of morphometric application. These findings are encouraging, however, given the limited sample size of this study, definitive conclusions should be drawn cautiously. This suggests that AI may serve as a potential supplementary tool for measuring structures on digital photographs, with the capacity to save time and reduce the risk of damaging delicate fixed specimens, though further validation with larger samples is recommended. These findings are consistent with the growing body of evidence supporting AI-assisted quantitative methods as effective alternatives to manual measurement in anatomical research [16,17]. In practice, AI measurement tools offer the additional advantage of enabling repeated, non-destructive measurements of archived digital photographs, a particular benefit when working with rare or irreplaceable specimens. The potential of AI to streamline morphometric workflows is particularly relevant in the context of large animal models such as sheep, used in applied neuroscience research where reproducible quantitative data is essential [21,22]. Virtual comparative anatomy platforms, which integrate multiple species models, further expand the possibilities for standardized digital morphometric study [26]. More broadly, Gayatri et al. [19] positions AI as a “valuable complementary tool” in veterinary anatomy, one that, when used optimally, may enhance rather than compete with traditional methods, a characterization that this study preliminarily supports for the specific domain of morphometric measurement, while emphasizing that such a role must be accompanied by expert oversight.

In contrast, the use of AI for labeling anatomical structures is currently insufficient for scientific research, and this study’s findings expose a significant and underappreciated limitation. The region-specific data presented in Table 2 reveal a pattern of failure that is both consistent and statistically striking across all three neuroanatomical domains examined. For the cerebrum, ChatGPT produced an error rate of 82.61% while Artlist recorded an error rate of 70.83%. These observations suggest that approximately three out of every four to five identification attempts were incorrect. For the cerebellum, the results were even more alarming: ChatGPT committed errors in 87.50% of attempts and Artlist produced an error rate of 92.86%, meaning that virtually every single cerebellar structure was mislabeled. Performance on brainstem structures was comparatively better but still clinically unacceptable, with ChatGPT exhibiting a 70.00% error rate and Artlist recording 38.89%. Considering all three regions combined, ChatGPT failed to correctly identify 33 out of 41 named structures (overall error rate: 80.49%), and Artlist failed on 50 out of 70 structures (overall error rate: 71.43%). These overall rates are not merely numerically high, but also statistically significant evidence of a systematic failure of AI in veterinary neuroanatomy. Given that a naive or random labeling strategy would be expected to yield substantially lower error rates, the near-ceiling error rates observed for the cerebrum and cerebellum indicate that current AI models are not simply uninformed but are actively applying incorrect, human-derived nomenclature to ovine neuroanatomical structures. These findings parallel and, in most cases, substantially exceed the error rates documented in human anatomy contexts. Hamasaki et al. [28] demonstrated that ChatGPT correctly identified only approximately 54% of specific anatomical structures in human anatomy assessments, while Hyeamang et al. [30] reported an overall AI accuracy of approximately 68% for image-based anatomical structure identification. Suárez et al. [29] similarly found that ChatGPT provided only 57.3% correct answers to binary clinical anatomy questions, confirming the unreliability of current models for accurate clinical nomenclature. Bolgova et al. [32] further evaluated ChatGPT’s performance in anatomy examinations, revealing significant gaps in factual anatomical knowledge. The fact that error rates in the present veterinary study substantially exceeded those reported even in human anatomy contexts reflects the comparatively impoverished training data available for veterinary species-specific neuroanatomy. The region-specific pattern of errors carries additional scientific significance and warrants careful interpretation. The exceptionally high cerebellar error rates (87.50% for ChatGPT; 92.86% for Artlist) are particularly informative: the ovine cerebellum contains lobular subdivisions, including the culmen, declive, folium vermis, tuber vermis, dorsal paraflocculus, ventral paraflocculus, and ansiform lobule, which have no direct human homologs under identical nomenclature. The failure of both AI tools to correctly identify these structures is therefore a direct and predictable consequence of absent species-specific training data, rather than a random or incidental finding. The cerebrum error rates (~71–83%) similarly reflect a systematic substitution of human gyral and sulcal terminology for the correct ovine equivalents: the most consistently observed error was applying the human term “central sulcus” instead of the correct veterinary term “cruciate sulcus” appeared repeatedly across both tools, providing unequivocal evidence that AI systems are applying a human-centric anatomical schema rather than retrieving species-appropriate knowledge. The comparatively lower Artlist error rate for brainstem structures (38.89%) suggests that brainstem landmarks, being more phylogenetically conserved across mammals, are slightly better represented in general-purpose AI training datasets. However, a failure rate approaching 40% on brainstem structures, which are critical for interpreting neurological deficits in clinical ovine and translational research settings, remains far beyond any acceptable scientific or clinical tolerance. Shamith et al. [33] documented systematic inaccuracies in AI-generated anatomical labels and image outputs, underscoring the general unreliability of current large language models for precise nomenclature tasks. Erbek et al. [31] provided a longitudinal perspective, noting that although accuracy trends in AI anatomy models have improved across model generations, the current performance remains insufficient for independent expert-level identification. Totlis et al. [35] characterized the potential role of ChatGPT and similar AI tools in anatomy education as supplementary aids, emphasizing that they should never substitute expert anatomical review. Critically, Choudhary et al. [16] offer a veterinary-specific analysis, noting that while AI holds enormous potential for the field, its limitations, particularly in species-specific nomenclature and the absence of validated veterinary training datasets, represent major obstacles to its reliable use. Bertin et al. [20] extended this concern to a research ethics framework, warning that uncritical AI adoption in veterinary research risks embedding systematic errors into the published literature if AI outputs are not rigorously validated by domain experts. For the purpose of methodological clarity, the errors observed in this study can be categorized into two main types: (1) locational errors, in which the AI identified a structure in the wrong anatomical area or region (e.g., misplacing the boundaries of the cerebellum or brainstem structures); and (2) nomenclature-based errors, in which the AI applied incorrect terminology derived from human anatomical convention rather than veterinary nomenclature (e.g., using the human term “central sulcus” instead of the correct ovine “cruciate sulcus”, or applying human-centric terms such as “superior” rather than the appropriate veterinary directional term “dorsal”). This distinction is important: nomenclature-based errors are a direct consequence of training data biased toward human anatomy, whereas locational errors may additionally reflect insufficient structural differentiation in the AI model. Both error types have significant implications for the reliability of AI tools in veterinary neuroanatomy research and education.

Furthermore, the clinical and scientific implications are substantial. Sheep are used as neurological disease models precisely because of the complexity and human-like organization of their brain anatomy [22,23,24], and inaccurate AI labeling in this context could propagate errors into downstream translational research and clinical interpretation. This study therefore calls upon the veterinary anatomy community to prioritize the development and curation of species-specific, expert-annotated neuroanatomical datasets that can be used to fine-tune existing AI models, a step that is essential for improving the accuracy, utility, and safety of AI tools in veterinary anatomical applications.

## 5. Conclusions

Artificial intelligence represents a promising yet currently limited tool in the study of veterinary anatomy. The present study provides preliminary evidence that AI tools including DeeVid AI (v1.0), Imageonline.io, ChatGPT, and Toolkit.artlist.io may be useful for reshaping 2D images into 3D models and for providing morphometric data comparable to traditional manual methods. However, for the identification and labeling of specific anatomical landmarks, AI currently falls critically short, often producing errors that could lead to clinical or scientific inaccuracies. Based on these findings, the use of these AI tools can be recommended only under human and expert supervision: AI-generated outputs should always be reviewed and validated by trained veterinary anatomists before being used in research, clinical, or educational contexts. This recommendation is particularly important for students, who increasingly rely on AI tools for self-directed study and for creating labeled images in assignments and presentations. The high error rates documented in this study serve as a caution that AI-generated anatomical labels may actively mislead learners, reinforcing incorrect terminology or misidentified structures, a risk that educators and academic supervisors must actively address. Future developments should focus on training AI models specifically on expert-annotated, species-specific veterinary anatomical datasets to improve nomenclature accuracy and reduce the risk of propagating human-centric anatomical errors into veterinary education and research.

## Figures and Tables

**Table 1:** Dorsal view morphometric measurements of the cerebrum (caliper vs. AI-derived value).

Dimension	Sample No.	Real (Caliper)	AI (Imageonline)
Length	1	7.50	7.40
	2	9.10	9.15
	3	8.90	8.89
	4	7.30	7.35
	5	9.60	9.59
	Mean ± SD	8.48 ± 1.02	8.48 ± 1.04
	*p*-value (Real vs. AI)		0.8914 (NS)
Width	1	5.00	5.15
	2	6.20	6.15
	3	5.40	5.43
	4	5.00	4.49
	5	6.30	6.35
	Mean ± SD	5.58 ± 0.634	5.51 ± 0.76
	*p*-value (Real vs. AI)		0.5982 (NS)

NS = Not significant (*p* > 0.05).

**Table 2:** Errors associated with AI-based labeling of anatomical structures in the sheep brain.

Brain Region (View)	N	ChatGPT			Artlist (toolkit.artlist.io)		
		Total Structures Named	Errors	Error Rate (%)	Total Structures Named	Errors	Error Rate (%)
Cerebrum (Dorsal view)	1	23	19	82.61%	24	17	70.83%
Cerebellum (Dorsal view)	1	8	7	87.50%	28	26	92.86%
Brainstem	1	10	7	70.00%	18	7	38.89%
Overall (Pooled)	—	41	33	80.49%	70	50	71.43%

N = specimen/image number. Error Rate (%) = (Errors ÷ Total Structures Named) × 100. Overall totals are pooled across all three regions.

## Data Availability

The original contributions presented in this study are included in the article. Further inquiries can be directed to the corresponding author.

## Abbreviations

The following abbreviations are used in this manuscript:

AI	Artificial Intelligence
CT	Computed Tomography
MRI	Magnetic Resonance Imaging
